# Prediction of gastric cancer risk: association between *ZBTB20* genetic variance and gastric cancer risk in Chinese Han population

**DOI:** 10.1042/BSR20202102

**Published:** 2020-09-24

**Authors:** Fei Bai, Ke Xiao

**Affiliations:** Department of Gastroduodenal Pancreas Surgery, Hunan Cancer Hospital, Changsha, 410013, Hunan, P.R. China

**Keywords:** Case-control study, gastric cancer, single nucleotide polymorphisms, ZBTB20

## Abstract

Background: Gastric cancer (GC) is a complex multifactorial disease. Previous studies have revealed genetic variations associated with the risk of gastric cancer. The purpose of the present study was to determine the correlation between single-nucleotide polymorphisms (SNPs) of *ZBTB20* and the risk of gastric cancer in Chinese Han population.

Methods: We conducted a ‘case–control’ study involving 509 GC patients and 507 healthy individuals. We selected four SNPs of *ZBTB20* (10934270 T/C, rs9288999 G/A, rs9841504 G/C and rs73230612 C/T), and used logistic regression to analyze the relationship between those SNPs and GC risk under different genetic models; multi-factor dimensionality reduction (MDR) was used to analyze the interaction of “SNP–SNP” in gastric cancer risk; ANOVA and univariate analysis were used to analyze the differences in clinical characteristics among different genotypes.

Results: Our results showed that *ZBTB20* rs9288999 is a protective factor for the risk of gastric cancer in multiple genetic models, of which the homozygous model is the most significant (OR = 0.48, *P*=0.0003); we also found that rs9288999 showed a significant correlation with reducing the risk of gastric cancer in different subgroups (BMI; age; gender; smoking or drinking status; adenocarcinoma); rs9841504 is associated with increased GC risk in the participants with BMI>24 kg/m^2^; rs9841504 and rs73230612 are certainly associated with clinical characteristics of platelet and carbohydrate antigen 242, respectively.

Conclusion: Our results suggest that *ZBTB20* rs9288999 may be important for reducing the risk of GC in the Chinese Han population.

## Introduction

Gastric cancer (GC) is considered to be a common gastrointestinal tumor in the world, the incidence is second only to lung cancer, breast cancer and colorectal cancer, and the survival rate is low [1]. The formation of gastric cancer is a complex process. As a multifactorial disease, gastric cancer is affected by both the environment and genetics [2]. A number of studies have confirmed that genetic factors are important causes of gastric cancer: Mohammad et al. suggested that people who are directly related to gastric cancer patients have a higher risk of gastric cancer than normal people [3]; Kaurah et al. found that 30–40% of patients with familial diffuse gastric cancer carry the *CDH1* gene mutation [4]. As we all know, single-nucleotide polymorphisms (SNPs) are the main form of genetic differences between individuals. With the development of molecular epidemiology and the improvement and application of genetic testing techniques, genetic polymorphisms associated with GC susceptibility have been identified in different populations [5–9].

*Zinc finger and BTB domain containing 20* (*ZBTB20*) protein is a member of the zinc finger protein subfamily. *ZBTB20* is a key regulator of α-fetoprotein expression in the adult liver. At the same time, *ZBTB20* has a high degree of homology with *BCL6* protein, and plays an important regulatory role in hematopoiesis, immune response and tumor development [10,11], and some studies have clarified the regulatory effect of *ZBTB20* on gastric migration, invasion and proliferation of GC [12]. Many scholars began to be interested in the association between the *ZBTB20* gene single-nucleotide polymorphism and gastric cancer susceptibility [13–15], but the results of these studies are not always same. More importantly, the incidence of gastric cancer is different in Eastern and Western countries, and even in different regions and different genders in China [16]. The above-mentioned studies suggest that individuals with different genetic backgrounds may have different susceptibility to gastric cancer. Therefore, it is necessary to expand the scope of the study to analyze the correlation between *ZBTB20* gene polymorphism and susceptibility of gastric cancer in different populations. Digging out biomarkers forgastric cancer susceptibility has potential biological and public health significance.

In summary, we conducted a correlation study between the *ZBTB20* SNPs and gastric cancer susceptibility in the Chinese Han population. In this study, 509 gastric cancer patients and 507 healthy individuals were collected at the same time and place. Combined with clinical data, we selected four sites rs10934270 T/C, rs9288999 G/A, rs9841504 G/C, rs73230612 C/T on*ZBTB20* gene for our study. Then, the correlation between *ZBTB20* SNPs and GC susceptibility in Chinese Han population was assessed; it will expand the association data between *ZBTB20* genetic variation and susceptibility of gastric cancer.

## Materials and methods

### Study subjects

The present study used a ‘case–control’ experimental design to assessthe correlation between the SNPs of the gene *ZBTB20* and the risk of GC in 1016 participants. These participants consisted of 509 patients with GC diagnosed at Hunan Cancer Hospital and 507 healthy individuals from the same hospital during the same period. We conducted interviews with the study subjects, and this work was carried out by professional doctors. After the interview, a complete questionnaire was formed, which included basic demographic and epidemiological information (age, gender, smoking, drinking, lymph node metastasis, pathological grade, medical history, etc.). Then, all participants provided blood samples for subsequent DNA extraction. Our study was approved by the Ethics Committee of Hunan Cancer Hospital, and all research work was carried out after the informed consents were obtained from all participants.

### Selection of SNPs

After consulting the relevant literature [13,17–22] and the data of the *ZBTB20* gene polymorphism in the database, we selected SNPs with minor allele frequency ≥ 5%. Finally, four SNPs of *ZBTB20* (rs10934270, rs9288999, rs9841504, rs73230612) were selected by us for the study.

### DNA extraction and genotyping

We carried out the extraction and purification of whole genomic DNA according to the experimental steps on the kit (GoldMag Co. Ltd. Xi’an, China) instructions. Subsequently, the extracted DNA was stored in a low temperature refrigerator (−80°C) until needed. The primers required for the study were designed by MassARRAY Assay Design software, Supplementary Table S1 summarized all the primers used for polymerase chain reaction (PCR) amplification and sequencing in this study. Then MassARRAY system (Agena, San Diego, CA, U.S.A.) was used for genotyping.

In order to reduce the influence of experimental operation errors on the research results an, we randomly selected 5% DNA samples for repeatability testing, and the experimental result repetition rate was >99%. The above steps can ensure the reliability and repeatability of the results of the present study.

### Statistical analysis

Differences in the demographic characteristics of the study carried out using SPSS software for χ^2^ test, the *P* value indicates whether it is statistically significant (*P*<0.05: statistically significant). After the testing whether the four candidate SNPs were in Hardy–Weinberg equilibrium (SPSS software), we conducted an overall analysis and stratified analysis (age, gender, smoking or drinking status and adenocarcinoma, etc.) of the association between *ZBTB20* gene polymorphism and GC risk. Using wild-type alleles as reference, plink 1.07 software was used to estimate multiple genetic models (codominant, dominant, recessive and logarithmic addition). The results of the present study are all estimated, based on the odds ratio (OR,) and 95% confidence interval (CI) derived from the analysis of the logistic regression model adjusted for age and gender (OR: relative risk; OR = 1: this factor has no effect on the occurrence of disease; OR < 1: reduce the risk of disease; OR > 1: increase the risk of disease). Then, we used multi-factor dimensionality reduction (MDR) to evaluate the candidate ‘SNP–SNP’ interaction in the risk of gastric cancer. Finally, we use one-way ANOVA to predict the differences in clinical characters of gastric cancer in different genotypes. All tests in the present study were two–sided tests, and *P*<0.05 was considered statistically significant.

## Results

### Research objects

The 1016 participants in the present study had no relationship in genetic. ‘Case–control’: a case group with an average age of 61.12 ± 11.33 years (male: 382 patients, proportion: 75%; female: 127 patients, proportion: 25%) and a control group with an average age of 61.35 ± 8.84 years (male: 379 healthy individuals, proportion: 75%; female: 128 healthy individuals, proportion: 25%). The results showed that there were no statistical differences in gender and age between the case group and the control group (Table 1). Table 1 summarized the demographic data of all participants, including age, gender, lymph node metastasis, adenocarcinoma, smoking and alcohol consumption, etc.

**Table 1:** Characteristics of patients with gastric cancer (GC) and healthy individuals

Characteristics		Cases	Control	*P*
		*n*=509	*n*=507	
Age (years)	Mean ± SD	61.12 ± 11.33	61.35 ± 8.84	0.712
	**>**60	279 (55%)	315 (62%)	
	≤60	230 (45%)	192 (38%)	
Gender	Male	382 (75%)	379 (75%)	0.942
	Female	127 (25%)	128 (25%)	
Lymph node metastasis	Yes	235 (46%)	–	
	No	97 (19%)	–	
Pathological grade	III and IV	239 (47%)	–	
	I and II	109 (21%)		
Adenocarcinoma	–	314 (62%)		
Smoking	Yes	233 (56%)	114 (22%)	
	No	270 (53%)	172 (34%)	
Drinking	Yes	133 (26%)	119 (23%)	
	No	357 (70%)	142 (28%)	
BMI (kg/m^2^)	BMI **>** 24	72 (14%)	183 (36%)	
	BMI ≤ 24	401 (79%)	170 (34%)	

BMI: body mass index.

### Information about genotyping and candidate SNPs

Four candidate SNPs (rs10934270, rs9288999, rs9841504, rs73230612) on *ZBTB20* were successfully genotyped. Detailed information about these four candidate SNPs was summarized in Table 2. All candidate SNPs were in HWE (*P*>5%), and they are all located in the intron region. The results of HaploReg indicate that the candidate SNPs in this study are regulated by a variety of factors, including: SiPhy cons; DNAse; Motifs changed; Selected eQTL hits and Enhancer histone marks etc.

**Table 2:** The basic information and HWE about the selected SNPs of *ZBTB20*

Gene	SNP ID	Role	Chr: Position	Alleles (A/B)	MAF		HWE (*P* value)	Haploreg 4.1
					Cases	Controls		
*ZBTB20*	rs10934270	Intron	3: 114384900	T/C	0.102	0.095	0.434	SiPhy cons; DNAse; Motifs changed; Selected eQTL hits
*ZBTB20*	rs9288999	Intron	3: 114429080	G/A	0.357	0.432	0.928	Enhancer histone marks; Motifs changed
*ZBTB20*	rs9841504	Intron	3: 114643917	G/C	0.145	0.134	0.848	Enhancer histone marks; Motifs changed; NHGRI/EBI GWAS hits
*ZBTB20*	rs73230612	Intron	3: 115131989	C/T	0.442	0.416	0.201	Motifs changed

HWE, Hardy–Weinberg equilibrium;

MAF, minor allele frequency;

SNP, single-nucleotide polymorphisms; *P*>0.05 indicates that the genotypes were in Hard–Weinberg equilibrium.

### Correlation assessment of ZBTB20 SNPs and GC risk (overall analysis)

The correlation between SNPs and GC risk under multiple genetic models was tested based on logistic regression, and the results were corrected by age and gender. The results showed that among the four candidate SNPs in the present study, only rs9288999 had a certain correlation with gastric cancer risk. Specifically: rs9288999 of *ZBTB20* is a protective factor (OR < 1) for the risk of gastric cancer under the allele model (G vs. A, OR = 0.73, CI = 0.61–0.87, *P*=0.001), homozygous model (GG vs. AA, OR = 0.48, CI = 0.33–0.71, *P*=0.0003), dominant model (GG-GA vs. AA, OR = 0.72, CI = 0.56–0.94, *P*=0.014), recessive model (GG vs. GA-AA, OR = 0.55, CI = 0.38–0.78, *P*=0.001) and additive model (OR = 0.72, CI = 0.60–0.87, *P*=0.0005). There is no correlation between the remaining three candidate SNPs and the risk of gastric cancer. The above results are summarized in Table 3.

**Table 3:** Analysis of the association between susceptibility of gastric cancer and single-nucleotide polymorphism of *ZBTB20*

SNP ID	Model	Genotype	Case	Control	Adjusted by age and gender	
					OR (95% CI)	*P*
**rs10934270**	Allele	T	104	96	1.09 (0.81–1.46)	0.571
		C	914	918	1.00	
	Genotype	TT	7	6	1.17 (0.39–3.52)	0.777
		TC	90	84	1.08 (0.78–1.50)	0.629
		CC	412	417	1.00	
	Dominant	TT-TC	97	90	1.09 (0.79–1.50)	0.595
		CC	412	417	1.00	
	Recessive	TT	7	6	1.16 (0.39–3.47)	0.796
		TC-CC	502	501	1.00	
	Log-additive	–	–	–	1.08 (0.81–1.45)	0.583
**rs9288999**	Allele	G	363	438	0.73 (0.61–0.87)	0.001*
		A	653	576	1.00	
	Genotype	GG	57	95	0.48 (0.33–0.71)	0.0003*
		GA	249	248	0.82 (0.62–1.07)	0.140
		AA	202	164	1.00	
	Dominant	GG-GA	306	343	0.72 (0.56–0.94)	0.014*
		AA	202	164	1.00	
	Recessive	GG	57	95	0.55 (0.38–0.78)	0.001*
		GA-AA	451	412	1.00	
	Log-additive	–	–	–	0.72 (0.60–0.87)	0.0005*
**rs9841504**	Allele	G	148	136	1.10 (0.85–1.41)	0.464
		C	870	878	1.00	
	Genotype	GG	14	8	1.76 (0.73–4.25)	0.208
		GC	120	120	1.01 (0.76–1.35)	0.935
		CC	375	379	1.00	
	Dominant	GG-GC	134	128	1.06 (0.80–1.40)	0.688
		CC	375	379	1.00	
	Recessive	GG	14	8	1.76 (0.73–4.23)	0.209
		GC-CC	495	499	1.00	
	Log-additive	–	–	–	1.10 (0.86–1.41)	0.466
**rs73230612**	Allele	C	449	422	1.11 (0.93–1.32)	0.241
		T	567	592	1.00	
	Genotype	CC	98	95	1.19 (0.83–1.69)	0.349
		CT	253	232	1.25 (0.94–1.65)	0.119
		TT	157	180	1.00	
	Dominant	CC-CT	351	327	1.23 (0.95–1.60)	0.121
		TT	157	180	1.00	
	Recessive	CC	98	95	1.04 (0.76–1.42)	0.809
		CT-TT	410	412	1.00	
	Log-additive	–	–	–	1.11 (0.93–1.32)	0.244

CI, confidence interval;

OR, odds ratio;

SNP, single-nucleotide polymorphisms;

*P*<0.05 indicates statistical significance.

### Correlation assessment of ZBTB20 SNPs and GC risk (subgroup analysis)

#### Age or gender

In participants ≤60 years old, rs9288999 of *ZBTB20* may reduce the risk of GC in multiple genetic models (allele model: OR = 0.59, *P*=0.0003; homozygous model: OR = 0.33, *P*=0.0002; dominant model: OR = 0.60, *P*=0.017, recessive model: OR = 0.39, *P*=0.0003 and log-additive model: OR = 0.60, *P*=0.0003). While among the male participants, the results showed that rs9288999 was significantly associated with reduction of gastric cancer risk in multiple genetic models (allele model: OR = 0.70, *P*=0.001; homozygous model: OR = 0.45, *P*<0.001; dominant model: OR = 0.68, *P*=0.012, recessive model: OR = 0.52, *P*=0.002 and log-additive model: OR = 0.69, *P*=0.001). The specific information is summarized in Table 4.

**Table 4:** The SNPs of *ZBTB20* associated with susceptibility of gastric cancer in the subgroup tests (age and gender)

SNP ID	Model	Genotype	Age, years				Gender			
			OR (95% CI)	*P*	OR (95% CI)	*P*	OR (95% CI)	*P*	OR (95% CI)	*P*
			>60		≤60		Female		Male	
**rs10934270**	Allele	T	1.24 (0.84–1.81)	0.280	0.91 (0.58–1.43)	0.681	1.01 (0.55–1.85)	0.978	1.11 (0.80–1.55)	0.530
		C	1.00		1.00		1.00		1.00	
	Genotype	TT	0.82 (0.15–4.58)	0.820	2.10 (0.40–11.11)	0.384	1.01 (0.06–16.46)	0.993	1.20 (0.36–3.98)	0.767
		TC	1.43 (0.93–2.21)	0.106	0.75 (0.44–1.25)	0.269	1.01 (0.52–1.96)	0.979	1.11 (0.76–1.62)	0.586
		CC	1.00		1.00		1.00		1.00	
	Dominant	TT-TC	1.39 (0.91–2.12)	0.130	0.82 (0.50–1.34)	0.422	1.01 (0.53–1.93)	0.978	1.12 (0.78–1.61)	0.552
		CC	1.00		1.00		1.00		1.00	
	Recessive	TT	0.77 (0.14–4.28)	0.763	2.2 (0.42–11.63)	0.353	1.01 (0.06–16.40)	0.994	1.18 (0.36–3.90)	0.789
		TC-CC	1.00		1.00		1.00		1.00	
	Log-additive	–	1.3 (0.88–1.93)	0.184	0.91 (0.59–1.41)	0.667	1.01 (0.55–1.85)	0.978	1.11 (0.80–1.53)	0.545
**rs9288999**	Allele	G	0.83 (0.65–1.05)	0.116	0.59 (0.45–0.78)	0.0003*	0.82 (0.57–1.17)	0.271	0.70 (0.57–0.87)	0.001*
		A	1.00		1.00		1.00		1.00	
	Genotype	GG	0.65 (0.38–1.12)	0.122	0.33 (0.18–0.59)	0.0002*	0.61 (0.29–1.30)	0.199	0.45 (0.28–0.70)	0.0005*
		GA	0.83 (0.58–1.18)	0.298	0.75 (0.48–1.17)	0.207	0.96 (0.56–1.65)	0.881	0.77 (0.57–1.06)	0.108
		AA	1.00		1.00		1.00		1.00	
	Dominant	GG-GA	0.79 (0.56–1.11)	0.171	0.60 (0.40–0.91)	0.017*	0.86 (0.51–1.44)	0.567	0.68 (0.51–0.92)	0.012*
		AA	1.00		1.00		1.00		1.00	
	Recessive	GG	0.73 (0.44–1.20)	0.213	0.39 (0.23–0.65)	0.0003*	0.62 (0.31–1.24)	0.179	0.52 (0.34–0.78)	0.002*
		GA-AA	1.00		1.00		1.00		1.00	
	Log-additive	–	0.81 (0.63–1.04)	0.105	0.60 (0.45–0.79)	0.0003*	0.82 (0.57–1.17)	0.266	0.69 (0.56–0.86)	0.001*
**rs9841504**	Allele	G	1.04 (0.75–1.43)	0.820	1.26 (0.83–1.90)	0.276	1.22 (0.74–2.00)	0.429	1.06 (0.79–1.42)	0.701
		C	1.00		1.00		1.00		1.00	
	Genotype	GG	1.16 (0.38–3.57)	0.797	6.47 (0.79–52.99)	0.082	2.62 (0.49–13.93)	0.259	1.50 (0.53–4.26)	0.450
		GC	1.11 (0.76–1.62)	0.587	1.00 (0.62–1.61)	0.993	1.04 (0.58–1.87)	0.890	1.00 (0.72–1.40)	0.998
		CC	1.00		1.00		1.00		1.00	
	Dominant	GG-GC	1.12 (0.77–1.61)	0.563	1.13 (0.71–1.80)	0.602	1.14 (0.65–2.00)	0.640	1.03 (0.74–1.43)	0.852
		CC	1.00		1.00		1.00		1.00	
	Recessive	GG	1.13 (0.37–3.45)	0.835	6.48 (0.79–52.89)	0.081	2.60 (0.49–13.74)	0.262	1.50 (0.53–4.25)	0.449
		GC-CC	1.00		1.00		1.00		1.00	
	Log-additive	–	1.10 (0.79–1.53)	0.566	1.25(0.83–1.88)	0.294	1.21 (0.75–1.96)	0.441	1.06 (0.79–1.42)	0.701
**rs73230612**	Allele	C	1.12 (0.89–1.41)	0.331	1.09(0.83–1.43)	0.542	0.86 (0.61–1.23)	0.412	1.21 (0.99–1.48)	0.068
		T	1.00		1.00		1.00		1.00	
	Genotype	CC	1.09 (0.68–1.75)	0.714	1.17 (0.66–2.05)	0.597	0.75 (0.37–1.53)	0.435	1.38 (0.92–2.08)	0.123
		CT	1.28 (0.88–1.86)	0.190	1.19 (0.77–1.85)	0.436	0.86 (0.50–1.50)	0.599	1.42 (1.03–1.96)	0.035
		TT	1.00		1.00		1.00		1.00	
	Dominant	CC-CT	1.22 (0.86–1.73)	0.256	1.18 (0.78–1.80)	0.427	0.83 (0.49–1.39)	0.481	1.41 (1.04–1.91)	0.586
		TT	1.00		1.00		1.00		1.00	
	Recessive	CC	0.95 (0.62–1.44)	0.801	1.05 (0.64–1.72)	0.858	0.82 (0.44–1.54)	0.543	1.13 (0.78–1.62)	0.526
		CT-TT	1.00		1.00		1.00		1.00	
	Log-additive	–	1.08 (0.85–1.35)	0.537	1.09 (0.83–1.45)	0.528	0.87 (0.61–1.23)	0.422	1.21 (0.98–1.47)	0.070

CI, Confidence interval;

OR, Odds ratio;

SNP: Single-nucleotide polymorphisms;

*P*<0.05 indicates statistical significance;

“–” indicates Log-additive model.

#### Smoking or drinking

The results show that rs9288999 was associated with reducing the risk of gastric cancer among participants who do not smoke or drink alcohol (OR < 1, *P*<0.05) in homozygous (GG vs. AA) and recessive models (GG vs. GA-AA). In this subgroup analysis, there was no association between the remaining three candidate SNPs and risk of GC. The specific information is shown in Table 5.

**Table 5:** The SNPs of *ZBTB20* associated with susceptibility of gastric cancer in the subgroup tests (smoking and drinking)

**SNP ID**	Model	Genotype	Smoking				Drinking			
			OR (95% CI)	*P*	OR (95% CI)	*P*	OR (95% CI)	*P*	OR (95% CI)	*P*
			Yes		No		Yes		No	
**rs10934270**	Allele	T	0.90 (0.56–1.46)	0.666	0.86 (0.54–1.36)	0.523	0.91 (0.54–1.56)	0.744	0.97 (0.61–1.55)	0.897
		C	1.00		1.00		1.00		1.00	
	Genotype	TT	1.30 (0.26–6.59)	0.754	0.29 (0.03–3.25)	0.315	2.04 (0.39–10.85)	0.401	0.42 (0.06–3.07)	0.395
		TC	0.76 (0.43–1.33)	0.335	0.93 (0.56–1.53)	0.763	0.72 (0.37–1.38)	0.317	1.08 (0.64–1.83)	0.775
		CC	1.00		1.00		1.00		1.00	
	Dominant	TT-TC	0.80 (0.46–1.38)	0.419	0.89 (0.54–1.45)	0.629	0.82 (0.44–1.51)	0.525	1.03 (0.62–1.72)	0.916
		CC	1.00		1.00		1.00		1.00	
	Recessive	TT	1.37 (0.27–6.92)	0.705	0.29 (0.03–3.29)	0.320	2.17 (0.41–11.48)	0.361	0.42 (0.06–3.03)	0.388
		TC-CC	1.00		1.00		1.00		1.00	
	Log-additive	–	0.87 (0.55–1.39)	0.565	0.85 (0.54–1.36)	0.505	0.95 (0.57–1.58)	0.829	0.98 (0.61–1.57)	0.923
**rs9288999**	Allele	G	0.78 (0.56–1.08)	0.132	0.77 (0.58–1.01)	0.059	0.75 (0.52–1.08)	0.120	0.79 (0.59–1.04)	0.095
		A	1.00		1.00		1.00		1.00	
	Genotype	GG	0.54 (0.27–1.07)	0.077	0.46 (0.26–0.82)	0.009*	0.49 (0.23–1.07)	0.072	0.54 (0.30–0.96)	0.035*
		GA	0.84 (0.51–1.39)	0.498	1.16 (0.76–1.78)	0.500	1.04 (0.60–1.8)	0.904	1.02 (0.66–1.58)	0.925
		AA	1.00		1.00		1.00		1.00	
	Dominant	GG-GA	0.75 (0.47–1.21)	0.237	0.92 (0.62–1.38)	0.690	0.86 (0.51–1.43)	0.555	0.87 (0.58–1.31)	0.513
		AA	1.00		1.00		1.00		1.00	
	Recessive	GG	0.59 (0.32–1.11)	0.101	0.42 (0.25–0.72)	0.002*	0.48 (0.24–0.99)	0.047	0.53 (0.31–0.90)	0.018*
		GA-AA	1.00		1.00		1.00		1.00	
	Log-additive	–	0.76 (0.54–1.05)	0.095	0.76 (0.57–1.00)	0.052	0.77 (0.53–1.10)	0.150	0.78 (0.59–1.04)	0.093
**rs9841504**	Allele	G	1.49 (0.88–2.51)	0.137	1.02 (0.70–1.47)	0.928	1.20 (0.69–2.06)	0.520	1.02 (0.69–1.50)	0.937
		C	1.00		1.00		1.00		1.00	
	Genotype	GG	/	0.999	2.00 (0.53–7.46)	0.305	/	0.999	1.48 (0.41–5.39)	0.551
		GC	1.43 (0.80–2.56)	0.234	0.87 (0.56–1.35)	0.542	1.06 (0.58–1.95)	0.840	0.92 (0.59–1.46)	0.732
		CC	1.00		1.00		1.00		1.00	
	Dominant	GG-GC	1.53 (0.86–2.73)	0.150	0.94 (0.62–1.44)	0.778	1.14 (0.62–2.06)	0.678	0.97 (0.62–1.50)	0.883
		CC	1.00		1.00		1.00		1.00	
	Recessive	GG	/	0.999	2.07 (0.56–7.69)	0.279	/	0.999	1.51 (0.42–5.47)	0.530
		GC-CC	1.00		1.00		1.00		1.00	
	Log-additive	–	1.59 (0.92–2.76)	0.100	1.02 (0.71–1.47)	0.914	1.21 (0.69–2.14)	0.512	1.02 (0.70–1.48)	0.937
**rs73230612**	Allele	C	1.09 (0.80–1.5)	0.581	1.15 (0.87–1.52)	0.326	1.37 (0.96–1.95)	0.080	1.12 (0.85–1.49)	0.417
		T	1.00		1.00		1.00		1.00	
	Genotype	CC	1.18 (0.62–2.23)	0.618	1.21 (0.67–2.17)	0.524	2.03 (0.97–4.25)	0.060	1.17 (0.66–2.05)	0.595
		CT	1.16 (0.68–1.98)	0.590	1.29 (0.85–1.97)	0.233	1.52 (0.83–2.77)	0.175	1.38 (0.90–2.13)	0.142
		TT	1.00		1.00		1.00		1.00	
	Dominant	CC-CT	1.16 (0.70–1.92)	0.552	1.27 (0.85–1.90)	0.237	1.65 (0.93–2.93)	0.085	1.32 (0.88–1.97)	0.177
		TT	1.00		1.00		1.00		1.00	
	Recessive	CC	1.07 (0.62–1.86)	0.799	1.05 (0.61–1.79)	0.868	1.54 (0.83–2.88)	0.172	0.98 (0.58–1.64)	0.932
		CT-TT	1.00		1.00		1.00		1.00	
	Log-additive	–	1.09 (0.79–1.50)	0.600	1.14 (0.86–1.51)	0.362	1.43 (0.99–2.07)	0.057	1.13 (0.86–1.50)	0.382

CI, Confidence interval;

OR, Odds ratio;

*P*<0.05 indicates statistical significance.

“–” indicates Log–additive model;

“/” indicates data missing.

#### BMI

The results are shown in Table 6: The rs9288999 of *ZBTB20* may reduce the risk of gastric cancer in the study population with BMI < 24 under the allele model (OR = 0.68, *P*=0.004), homozygous model (OR = 0.41, *P*=0.001), recessive model (OR = 0.46, *P*=0.002) and additive model (OR = 0.67, *P*=0.004); on the contrary, rs9841504 may increase the risk of gastric cancer in the study population with BMI> 24 under the homozygous model (OR = 11.9, *P*=0.028) and recessive model (OR = 12.29, *P*=0.026).

**Table 6:** The SNPs of *ZBTB20* associated with susceptibility of gastric cancer in the subgroup tests (BMI)

SNP ID	Model	genotype	BMI			
			OR (95% CI)	*P*	OR (95% CI)	*p*
			<24		>24	
**rs10934270**	Allele	T/C	0.87 (0.58–1.30)	0.490	1.26 (0.66–2.41)	0.490
	Homozygote	TT/CC	0.78 (0.19–3.19)	0.726	2.90 (0.18–47.36)	0.456
	Heterozygote	TC	0.83 (0.52–1.32)	0.425	1.17 (0.56–2.42)	0.680
	Dominant	TT-TC/CC	0.82 (0.52–1.29)	0.396	1.22 (0.60–2.48)	0.584
	Recessive	TT/TC-CC	0.80 (0.20–3.30)	0.762	2.82 (0.17–45.89)	0.467
	Log-additive	–	0.84 (0.56–1.26)	0.401	1.26 (0.65–2.43)	0.500
**rs9288999**	Allele	G/A	0.68 (0.53–0.89)	0.004*	0.80 (0.54–1.19)	0.265
	Homozygote	GG/AA	0.41 (0.24–0.71)	0.001*	0.57 (0.24–1.39)	0.220
	Heterozygote	GA	0.84 (0.56–1.26)	0.389	0.77 (0.42–1.41)	0.391
	Dominant	GG-GA/AA	0.70 (0.48–1.03)	0.072	0.72 (0.40–1.29)	0.269
	Recessive	GG/GA-AA	0.46 (0.28–0.74)	0.002*	0.68 (0.30–1.52)	0.342
	Log-additive	–	0.67 (0.51–0.88)	0.004*	0.76 (0.50–1.15)	0.198
**rs9841504**	Allele	G/C	1.41 (0.95–2.10)	0.087	1.43 (0.85–2.40)	0.180
	Homozygote	GG/CC	3.56 (0.44–29.1)	0.236	11.9 (1.31–108.82)	0.028*
	Heterozygote	GC	1.32 (0.84–2.05)	0.227	0.87 (0.45–1.69)	0.688
	Dominant	GG-GC/CC	1.38 (0.89–2.14)	0.149	1.11 (0.60–2.04)	0.744
	Recessive	GG/GC-CC	3.35 (0.41–27.33)	0.259	12.29 (1.36–111.20)	0.026*
	Log-additive	–	1.40 (0.93–2.10)	0.105	1.35(0.80–2.28)	0.267
**rs73230612**	Allele	C/T	1.16 (0.90–1.50)	0.256	0.98 (0.67–1.45)	0.937
	Homozygote	CC/TT	1.37 (0.80–2.34)	0.248	0.89 (0.38–2.06)	0.777
	Heterozygote	CT	1.13 (0.75–1.70)	0.558	1.39 (0.74–2.61)	0.302
	Dominant	CC-CT/TT	1.19 (0.81–1.75)	0.374	1.24 (0.68–2.26)	0.482
	Recessive	CC/CT-TT	1.27 (0.79–2.04)	0.319	0.72 (0.34–1.51)	0.385
	Log–additive	–	1.16 (0.90–1.51)	0.251	1.00 (0.67–1.48)	0.987

CI, Confidence interval;

OR, Odds ratio;

SNP: Single-nucleotide polymorphisms;

*P*<0.05 indicates statistical significance;

“–” indicates Log-additive model.

#### Adenocarcinoma

In gastric cancer patients with adenocarcinoma, rs9288999 is a protective factor for the risk of gastric cancer in multiple genetic models (OR < 1, *P*<0.05), such as allele model (OR = 0.72, *P*=0.002), homozygous model (OR = 0.52, *P*=0.004), heterozygous model (OR = 0.72, *P*=0.037), dominant model (OR = 0.67, *P*=0.006), recessive model (OR = 0.63, *P*=0.024) and additive model (OR = 0.72, *P*=0.002). The specific information is summarized in Table 7.

**Table 7:** The SNPs of *ZBTB20* associated with susceptibility of gastric cancer in the subgroup tests (adenocarcinoma)

SNP ID	Model	genotype	Adenocarcinoma (patients with GC)			
			Case	Control	OR (95% CI)	*P*
			Yes	No		
**rs10934270**	Allele	T/C	60	96	1.01 (0.72–1.42)	0.954
	Homozygote	TT/CC	5	6	1.30 (0.39–4.32)	0.667
	Heterozygote	TC	50	84	0.95 (0.65–1.40)	0.804
	Dominant	TT-TC/CC	55	90	0.98 (0.67–1.41)	0.898
	Recessive	TT/TC-CC	5	6	1.31 (0.40–4.35)	0.656
	Log-additive	–	–	–	1.00 (0.72–1.39)	0.996
**rs9288999**	Allele	G/A	222	438	0.72 (0.59–0.89)	0.002*
	Homozygote	GG/AA	40	95	0.52 (0.34–0.81)	0.004*
	Heterozygote	GA	142	248	0.72 (0.53–0.98)	0.037*
	Dominant	GG-GA/AA	182	343	0.67 (0.50–0.89)	0.006*
	Recessive	GG/GA-AA	40	95	0.63 (0.42–0.94)	0.024*
	Log-additive	–	–	–	0.72 (0.59–0.89)	0.002*
**rs9841504**	Allele	G/C	93	136	1.12 (0.84–1.49)	0.427
	Homozygote	GG/CC	8	8	1.68 (0.62–4.53)	0.309
	Heterozygote	GC	77	120	1.07 (0.77–1.49)	0.697
	Dominant	GG-GC/CC	85	128	1.11 (0.80–1.52)	0.538
	Recessive	GG/GC-CC	8	8	1.65 (0.61–4.44)	0.322
	Log–additive	–	–	–	1.13 (0.85–1.50)	0.405
**rs73230612**	Allele	C/T	282	422	1.15 (0.94–1.41)	0.173
	Homozygote	CC/TT	64	95	1.28 (0.86–1.92)	0.224
	Heterozygote	CT	154	232	1.26 (0.91–1.73)	0.164
	Dominant	CC-CT/TT	218	327	1.26 (0.93–1.71)	0.128
	Recessive	CC/CT-TT	64	95	1.12 (0.79–1.60)	0.523
	Log-additive	–	–	–	1.15 (0.94–1.40)	0.174

CI, Confidence interval;

OR, Odds ratio;

SNP: Single-nucleotide polymorphisms;

*P*<0.05 indicates statistical significance;

“–” indicates Log–additive model.

In addition, we also divided the gastric cancer cases in the present study according to the pathological grade (I and II vs. III and IV) and whether the lymph nodes metastasized. No association have been found between candidate SNPs and gastric cancer risk (Supplementary Table S2).

### MDR analysis

**Figure 1:**
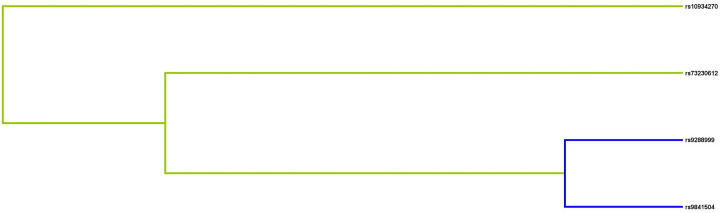
Dendrogram analysis of SNP–SNP interaction The colors in the tree diagram represent synergy (yellow) or redundancy (blue).

**Figure 2:**
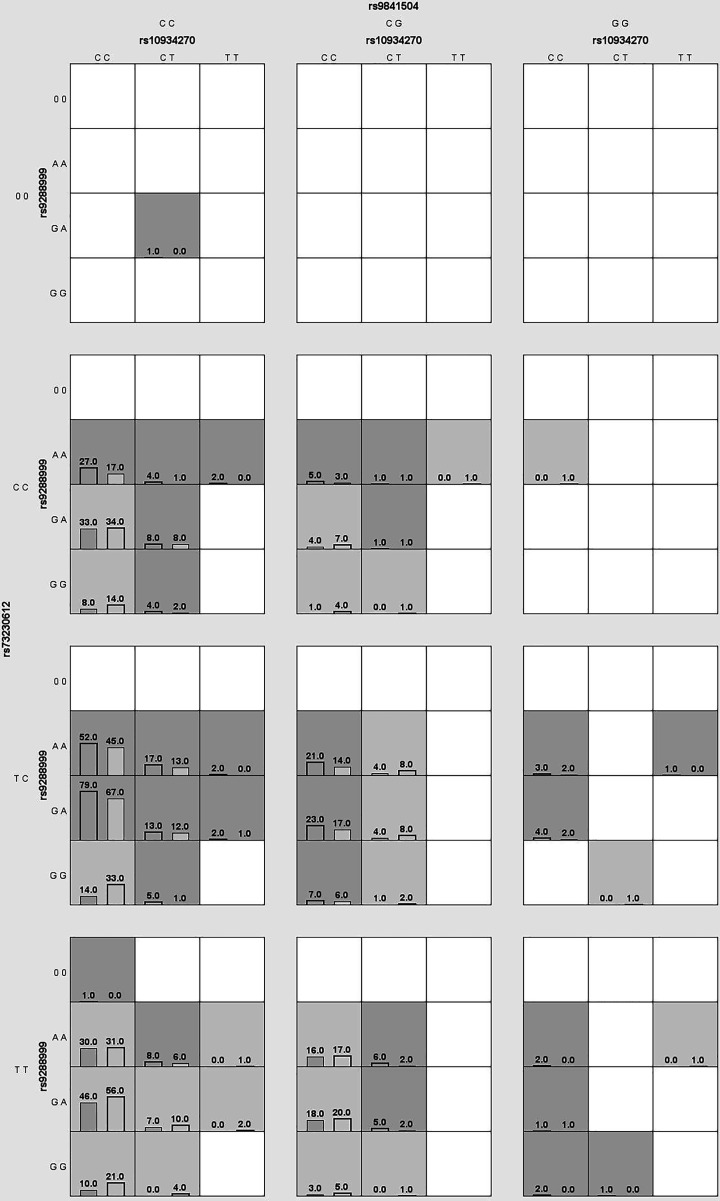
Multifactor dimensionality reduction (MDR) analysis for SNPs (10934270, rs9288999, rs9841504 and rs73230612) of *ZBTB20* interaction In each box, the left bar represents cases and the right bar represents controls. The light gray boxes indicate the low risk of gastric cancer and dark gray boxes indicate the high risk, the empty boxes mean no data.

Subsequently, MDR analysis was used to assess the interaction of ‘SNP–SNP’. Figure 1 shows the interaction between the four candidate SNPs of *ZBTB20*. The blue line indicates that these four SNPs may have a redundant role in regulating the risk of diabetes. All experimental results have been shown in Table 8. The best unit point model for predicting the risk of gastric cancer is: rs9288999 (testing accuracy = 0.521, CVC = 10/10, *P*=0.0006); the two-site model is:rs9288999, rs73230612 (testing accuracy = 0.535, CVC = 10/10, *P*=0.0003); the three-site model is: rs10934270, rs9288999 and rs73230612 (testing accuracy = 0.500, CVC = 7/10, *P*<0.0001); the four-site model is: rs10934270, rs9288999, rs9841504 and rs73230612 (testing accuracy = 0.517, CVC = 10/10, *P*<0.0001). Figure 2 shows the interaction of ‘SNP–SNP’ in different combinations of sites, among them, the light gray grid represents a low risk of gastric cancer, the darker gray grid represents a higher risk of gastric cancer, and the unfilled grid represents no data. Therefore, we can conclude that the impact of these four candidate SNPs of *ZBTB20* on the risk of gastric cancer may be interdependent.

**Table 8:** SNP–SNP interaction models analyzed by the MDR method

Model	Training Bal. Acc	Testing Bal. Acc	OR (95% CI)	*P* value	CVC
rs9288999	0.540	0.521	1.86 (1.30–2.65)	**0.0006**	10/10
rs9288999, rs73230612	0.557	0.535	1.60 (1.24–2.05)	**0.0003**	10/10
rs10934270, rs9288999, rs73230612	0.569	0.500	1.72 (1.33–2.22)	**<0.0001**	7/10
rs10934270, rs9288999, rs9841504, rs73230612	0.586	0.517	1.96 (1.52–2.51)	**<0.0001**	10/10

Bal. Acc., balanced accuracy;

CVC, cross-validation consistency;

MDR, multifactor dimensionality reduction;

OR, odds ratio;

95% CI, 95% confidence interval.

*P* values were calculated using χ2 tests;

*P*<0.05 indicates statistical significance.

### Differences in clinical indicators under different genotypes

Finally, we also evaluated the correlation between the four candidate SNPs polymorphisms (rs10934270, rs9288999, rs9841504, rs73230612) and clinical indicators of gastric cancer patients. These clinical indicators include carcinoembryonic antigen (CEA), tumor necrosis factor (TNF), carbohydrate antigen 50 (CA50), carbohydrate antigen 19-9 (CA19-9), carbohydrate antigen 242 (CA242), white blood cells (WBC), hemoglobin (HGB) and platelet (PLT). The results showed (Table 9 and Supplementary Table S3): The rs9841504 of *ZBTB20* had a potential association with the content of PLT (*P*=0.048), while the rs73230612 had a significant association with CA242 (*P*=0.005).

**Table 9:** Clinical characteristics of patients based on the genotypes of selected SNPs

Characteristics	rs9841504				rs73230612			
	CC	CG	GG	*P*	TT	TC	CC	*P*
CEA	17.28 ± 10.59	16.99 ± 11.16	14.06 ± 3.49	0.637	17.38 ± 8.85	16.24 ± 9.34	18.94 ± 15.26	0.230
TNF (fmol/ml)	0.90 ± 0.07	1.39 ± 4.30	0.88 ± 0.06	0.196	0.88 ± 0.07	1.12 ± 2.92	0.90 ± 0.07	0.569
CA50 (U/ml	7.48 ± 11.94	7.74 ± 12.76	6.05 ± 7.78	0.916	8.35 ± 13.58	7.02 ± 11.04	7.45 ± 12.01	0.681
CA19–9 (U/ml)	45.57 ± 89.00	55.45 ± 107.75	20.19 ± 8.30	0.491	51.65 ± 91.31	44.68 ± 94.12	44.63 ± 90.63	0.827
CA242 (KU/ml)	16.48 ± 30.97	14.11 ± 27.37	5.47 ± 7.72	0.493	23.51 ± 42.37	11.39 ± 19.71	12.87 ± 23.18	0.005*
WBC (L)	6.27 ± 5.92	7.83 ± 6.67	3.83 ± 2.13	0.293	8.14 ± 8.17	5.96 ± 5.45	6.16 ± 3.66	0.127
HGB (g/l)	102.76 ± 22.62	111.02 ± 28.04	93.5 ± 37.03	0.108	105.38 ± 26.76	102.33 ± 22.16	108.7 ± 26.79	0.417
PLT (L)	189.64 ± 94.02	229.40 ± 132.05	307.00 ± 89.10	0.048*	203.16 ± 99.62	207.62 ± 117.15	187.68 ± 94.63	0.669

CA50, carbohydrate antigen 50;

CA19-9, carbohydrate antigen 19-9;

CA242, carbohydrate antigen 242;

CEA, carcinoembryonic antigen;

HGB, hemoglobin;

PLT, platelet;

TNF, tumor necrosis factor;

WBC, white blood cells.

## Discussion

Gastric cancer is the result of multiple gene–environment interactions, and a single gene plays a smaller role in it [23]. It is estimated that only a small part of the tumor susceptibility areas/sites found in GWAS can explain the occurrence of tumors. Among them, prostate cancer can reach 15%, while in breast and colorectal cancer, only 5% and 4%, even less in gastric cancer [24]. Therefore, it is still a long-term and arduous task to carry out more researches on the association of gastric cancer risk to identify more unknown susceptible areas/sites. The incidence of gastric cancer varies among different societies, East Asia, Central America, South America and Eastern Europe have higher incidence, while Africa and North America have lower incidence [1,25]. There are differences in the incidence of gastric cancer even between north and south of China [16]. Wherefore, it is very necessary to conduct gastric cancer association studies in different populations. These research results are of great significance for us to understand the susceptibility mechanism of gastric cancer, to explore the pathogenesis of gastric cancer, the risk prediction and screening of high-risk groups, and to guide the individualized treatment of gastric cancer.

Some cancer-related studies have found that NF-Κb (nf-kappa b) may cause oncogenesis, which is carried out by induction of genes encoding proteins. These proteins are related to invasion, migration and inhibition of cell apoptosis [26,27]. Liu et al. found that *ZBTB20* can promote the activation of NF-κB through inhibiting IjBa gene transcription or regulating protein expression [28]. And there were studies showed that overexpression of *ZBTB20* can promote the proliferation, migration and invasion of gastric cancer cells, which may be regulated by the IκBα/NF-κB signaling pathway [12]. The above research results suggested that *ZBTB20* may be a target for gastric cancer prevention and treatment. For genetics field, some studies have pointed out that genetic mutations and gene polymorphisms are the main reasons for the differences in the gastric risk among individuals [29,30]. Some polymorphism sites are significantly associated with gastric cancer risk have been reported [31–34], but there was no research about the association between *ZBTB20* and the risk of gastric cancer in Chinese Han population.

Therefore, in our study, the Chinese Han population was used as the research object to conduct a ‘case–control’ study, then we analyzed the correlation between *ZBTB20* gene polymorphisms (rs10934270, rs9288999, rs9841504, rs73230612) and gastric cancer risk. The results of our study showed that *ZBTB20* rs9288999 was significantly associated with reducing the risk of gastric cancer among the study population, whether in the overall analysis or stratified analysis. Dong et al. and Yusefi et al. proposed that genetic polymorphisms may affect disease risk by regulating the expression of certain genes [29,30]. Combining the results of our study, we speculated that the polymorphic site ‘rs9288999’ may have regulated the expression of *ZBTB20* through the IκBα/NF-κB signaling pathway, which made rs9288999 showed a significant association with reduction of gastric cancer risk in the Chinese Han population. But this complicated process still needs more comprehensive research to verify. Nevertheless, as far as we know, the present study is the first to find evidence of a correlation between *ZBTB20* rs9288999 and gastric cancer risk. At the same time, the results of our study also provided new genetic-related research directions for gastric cancer prevention and treatment in the future.

Since gastric cancer is a multifactorial disease, the prerequisite for prevention is to accurately identify and manage the risk factors or potential causes of gastric cancer [35]. Studies have found evidence that people who smoke and drink alcohol may have a higher risk of gastric cancer than those who do not smoke or drink alcohol [36–38]. Lope et al. found that the case group (patients with gastric cancer) was significantly older than the control group [39]. Studies have shown that gender factors may also play a role in gastric cancer risk [40]. We got similar results and found that there was a significant correlation between *ZBTB20* rs9288999 and reducing the gastric cancer risk in participants who are non-smokers (OR = 0.46, *P*=0.009), non-drinking (OR = 0.54, *P*=0.035), age ≤ 60 years (OR = 0.33, *P*=0.0002) and male participants (OR = 0.45, *P*=0.0005). Based on previous studies and our study, the cause of the above results may be the interaction between environmental factors and genetic polymorphisms. What’s more, *ZBTB20* rs9288999 may play a certain role in that.

In addition, exploring the interaction between SNP–SNP can also help us to discover potential risk factors for the incidence of gastric cancer. Therefore, MDR was used to explore the interaction between the four candidate SNPs. The results showed that for gastric cancer risk, rs10934270, rs9288999, rs9841504 and rs73230612 showed a strong interaction.

In the correlation analysis between clinical indicators and gastric cancer risk, we also found that the *ZBTB20* polymorphic site rs9841504 and platelet showed a certain significant correlation (*P*=0.048). Platelet involves in cancer growth and metastasis is a long-term concept [41], and studies have shown that the ratio of platelet to lymphocyte and CA242 may be convenient biomarkers for gastric cancer prognosis [42,43]. Therefore, our results suggested that rs9841504 may play a certain role in the influence of platelets on the occurrence and development of gastric cancer. However, due to the limitation of sample size and ethnicity, this result can only be used as a reminder. It requires deeper research to be accurately verified.

This study provides data supplements for the association between the *ZBTB20* gene polymorphisms and the risk of gastric cancer in the Chinese Han population, and concludes that there may have certain association between the two. However, we must face the fact that our research has certain limitations, which is not only for the confirmation of results but also for new discoveries, a large sample size is indeed necessary. Currently, the genetic regions/sites discovered are only a small part, and there are more genetic susceptible sites/regions waiting to be discovered. With the discovery of susceptibility sites for gastric cancer in the future, we will have a more comprehensive understanding of the genetics of gastric cancer.

## Conclusion

In summary, our study is the first to find that the rs9288999 of *ZBTB20* has a potential association with reducing the risk of gastric cancer in the Chinese Han population. It provides new data supplement for the study of the association between *ZBTB20* gene polymorphism and gastric cancer risk.

## Supplementary Material

Supplementary Tables S1-S3

## Data Availability

The datasets used and analyzed in the current study are available from the corresponding author on reasonable request.

## Abbreviations

CA50: carbohydrate antigen 50
CA19-9: carbohydrate antigen 19-9
CA242: carbohydrate antigen 242
CEA: carcinoembryonic antigen
HGB: hemoglobin
GC: gastric cancer
PLT: platelet
TNF: tumor necrosis factor
WBC: white blood cells
