# Supplementary material for: Prediction of gastric cancer risk: association between ZBTB20 genetic variance and gastric cancer risk in Chinese Han population
Source: Biosci Rep. 2020 Sep 24;40(9):BSR20202102. doi: 10.1042/BSR20202102 (PMC7517264; doi:10.1042/BSR20202102)
Supplement: Supplementary Tables S1-S3 [file BSR-2020-2102_supp.pdf]

Supplemental table 1 The primers for *ZBTB20* gene amplification and sequencing.

SNP	1st-PCR primer	2nd-PCR primer	UEP-DIR	UEP-SEQ
rs10934270	ACGTTGGATGTGCCATCACTGTATGTGTGC	ACGTTGGATGGAAATTAACCAGATCGGCGG	F	GCATTTGATGTATATATTTCACA
rs9288999	ACGTTGGATGATTGCTGGGTAGTAAAAAG	ACGTTGGATGTGAATCTAGTCCGTGGCTTC	R	atgtTAGTGAAAATGCTACAACAC
rs9841504	ACGTTGGATGGAATCTCACTGTTAAGGAGC	ACGTTGGATGACATTTTGCCCGTACCTCTC	F	TCCCTTTTTCCCTATATATTTTTA
rs73230612	ACGTTGGATGCTAGGCCATGACCATGTTGT	ACGTTGGATGATGGTAACGAAAGGCAAGCA	R	CAAGCAAATATGAAAAAGCAATGA

SNP, single nucleotide polymorphism;

UEP-DIR, Uniqueextension primer-direction;

UEP-SEQ, Unique extension primer-sequencing.

Supplemental table 2 The correlation between SNPs and gastric cancer sensitivity in subgroups(pathological grade and lymph node metastasis).

			**Pathological grade**				**Lymph node metastasis**			
**SNP ID**	**Model**	**Genotype**	**Case Ⅲ&Ⅳ**	**Control Ⅰ&Ⅱ**	**OR (95% CI)**	***p***	**Case Yes**	**Control No**	**OR (95% CI)**	***p***
**rs10934270**	Allele	T	52	21	1.15(0.67-1.95)	0.619	49	19	1.07(0.61-1.87)	0.807
		C	426	197	1.00		421	175	1.00	
	Genotype	TT	4	2	1.01(0.18-5.67)	0.993	3	1	1.32(0.13-13.01)	0.810
		TC	44	17	1.31(0.70-2.44)	0.403	43	17	1.13(0.60-2.12)	0.699
		CC	191	90	1.00		189	79	1.00	
	Dominant	TT-TC	48	19	1.27(0.70-2.32)	0.428	46	18	1.14(0.62-2.11)	0.668
		CC	191	90	1.00		189	79	1.00	
	Recessive	TT	4	2	0.96(0.17-5.40)	0.966	3	1	1.30(0.13-12.70)	0.824
		TC-CC	235	107	1.00		232	96	1.00	
	Log-additive	-	-	-	1.20(0.71-2.04)	0.495	-	-	1.14(0.65-1.99)	0.655
**rs9288999**	Allele	G	170	78	1.00(0.71-1.39)	0.987	165	65	1.08(0.76-1.54)	0.667
		A	306	140	1.00		303	129	1.00	
	Genotype	GG	26	16	0.77(0.37-1.59)	0.478	27	10	1.13(0.50-2.54)	0.778
		GA	118	46	1.27(0.77-2.07)	0.349	111	45	1.07(0.64-1.76)	0.807
		AA	94	47	1.00		96	42	1.00	
	Dominant	GG-GA	144	62	1.14(0.72-1.81)	0.588	138	55	1.08(0.66-1.74)	0.766
		AA	94	47	1.00		96	42	1.00	
	Recessive	GG	26	16	0.68(0.35-1.34)	0.265	27	10	1.09(0.50-2.36)	0.831
		GA-AA	212	93	1.00		207	87	1.00	
	Log-additive	-	-	-	0.98(0.69-1.37)	0.885	-	-	1.06(0.74-1.53)	0.746
**rs9841504**	Allele	G	66	38	0.76(0.49-1.17)	0.214	67	35	0.76(0.48-1.18)	0.219
		C	412	180	1.00		403	159	1.00	
	Genotype	GG	8	5	0.64(0.20-2.05)	0.452	5	5	0.37(0.10-1.33)	0.128
		GC	50	28	0.74(0.43-1.26)	0.266	57	25	0.88(0.50-1.52)	0.641
		CC	181	76	1.00		173	67	1.00	
	Dominant	GG-GC	58	33	0.72(0.43-1.20)	0.209	62	30	0.79(0.47-1.33)	0.379
		CC	181	76	1.00		173	67	1.00	
	Recessive	GG	8	5	0.69(0.22-2.20)	0.530	5	5	0.38(0.11-1.37)	0.139
		GC-CC	231	104	1.00		230	92	1.00	
	Log-additive	-	-	-	0.76(0.50-1.16)	0.208	-	-	0.76(0.49-1.17)	0.212

**rs73230612**	Allele	C	210	96	1.00(0.73-1.39)	0.984	206	89	0.93(0.66-1.30)	0.661
		T	266	122	1.00		262	105	1.00	
	Genotype	CC	45	21	1.04(0.53-2.01)	0.919	43	22	0.84(0.43-1.66)	0.621
		CT	120	54	1.08(0.64-1.82)	0.775	120	45	1.14(0.65-1.98)	0.647
		TT	73	34	1.00		71	30	1.00	
	Dominant	CC-CT	165	75	1.07(0.65-1.75)	0.798	163	67	1.04(0.62-1.75)	0.879
		TT	73	34	1.00		71	30	1.00	
	Recessive	CC	45	21	0.99(0.55-1.77)	0.965	43	22	0.78(0.44-1.40)	0.401
		CT-TT	193	88	1.00		191	75	1.00	
	Log-additive	-	-	-	1.03(0.74-1.43)	0.885	-	-	0.94(0.67-1.32)	0.704

Supplemental table 3 Clinical characteristics of patients based on the genotypes of selected SNPs.

Characteristics	rs10934270				rs9288999			
	**TT**	**TC**	**CC**	***p***	**AA**	**AG**	**GG**	***p***
CEA	16.83 ± 9.26	15.8 ± 8.07	17.41 ± 11.07	0.582	17.56 ± 11.83	16.9 ± 9.31	16.66 ± 12.06	0.846
TNF (fmol/ml)	0.87 ± 0.07	0.89 ± 0.08	1.04 ± 2.3	0.877	1.19 ± 3.29	0.89 ± 0.06	0.9 ± 0.09	0.459
CA50 (U/ml	13.52 ± 15.32	8.29 ± 13.96	7.2 ± 11.5	0.385	8.47 ± 13.86	7.3 ± 10.98	4.5 ± 8.38	0.251
CA19-9 (U/ml)	75.59 ± 117.45	53.91 ± 105.74	45.01 ± 89.03	0.607	52.15 ± 105.46	47.87 ± 90.62	24.71 ± 21.62	0.326
CA242 (KU/ml)	12.06 ± 16.71	16.33 ± 28	15.53 ± 30.41	0.949	13.52 ± 27.15	17.83 ± 32.05	12 ± 26.79	00385
WBC(L)	5.74 ± 2.72	6.27 ± 4.06	6.73 ± 6.52	0.905	7.64 ± 8.04	6.18 ± 4.32	5 ± 2.76	0.139
HGB(g/L)	118.33 ± 29.67	109.46 ± 27.85	103.36 ± 23.78	0.304	104.93 ± 26.32	105.18 ± 24.56	101.92 ± 19.9	0.845
PLT(L)	166 ± 110.31	211.25 ± 89.15	200.38 ± 110.29	0.807	213.55 ± 118.84	202.94 ± 103.18	161.24 ± 65.01	0.149

CEA, carcinoembryonic antigen;

TNF, tumor necrosis factor;

CA50, carbohydrate antigen 50;

CA19-9, carbohydrate antigen 19-9;

CA242, carbohydrate antigen 242;

WBC, white blood cells;

HGB, hemoglobin;

PLT, platelet.
